# The predictive model of intra-arrest blood markers for the sustained return of spontaneous circulation in patients with out-of-hospital cardiac arrest

**DOI:** 10.3389/fmed.2026.1904268

**Published:** 2026-10-13

**Authors:** Shurong Xu, Sa Wang, Shuaishuai Zhou, Yajie Liu, Jue Fang, Fenfang Zhan, Gaoxing Lin, Danping Yan, Meiling Wang, Yuwei Wang

**Affiliations:** 1 Department of Nursing, The Second Affiliated Hospital, Zhejiang University School of Medicine, Hangzhou, China; 2 Department of Emergency Medicine, The Second Affiliated Hospital, Zhejiang University School of Medicine, Hangzhou, China

**Keywords:** blood analysis, cardiopulmonary resuscitation, emergency department, internal environment, ROSC (return of spontaneous circulation)

## Abstract

**Objective:**

This study aimed to investigate the predictive value of intra-arrest blood markers, including blood routine, blood biochemistry, and coagulation profile, during cardiopulmonary resuscitation (CPR) for the return of spontaneous circulation (ROSC) in patients with out-of-hospital cardiac arrest (OHCA).

**Methods:**

A retrospective cohort of patients who experienced OHCA and admitted to the emergency department of a tertiary hospital in Zhejiang Province, China, between January 2021 and December 2025 was assembled. Their clinical and demographic data were collected, and missing values were addressed using multiple imputation. Physiologic laboratory markers obtained during resuscitation were compared between the sustained ROSC and non-ROSC groups. Candidate predictors were selected based on clinical relevance, previous literature, availability at the intended prediction time point, and univariable screening, with age and gender retained as prespecified demographic covariates. Model discrimination for the prediction of sustained ROSC was evaluated using a receiver operating characteristic analysis and the area under the curve (AUC). Internal validation was performed using bootstrap resampling to assess model stability and quantify potential overfitting.

**Results:**

A total of 283 patients were included, and among them, 94 (33.22%) achieved sustained ROSC. Missing data were handled using multiple imputation (*m* = 20). In univariable analyses, the sustained ROSC group had higher PCO_2_, platelet count, white blood cell count, and absolute lymphocyte count; higher admission glucose, fibrinogen, and serum calcium levels; shorter chest compression duration and prothrombin time; and lower epinephrine dose, serum potassium level, and international normalized ratio than the non-sustained ROSC group (all *P* < 0.05). A multivariable logistic regression model incorporating age; gender; white blood cell count; platelet count; blood PCO_2_ measured during CPR; capillary blood glucose level on admission; and fibrinogen, serum potassium, and serum calcium levels yielded a mean apparent AUC of 0.832 across the imputed datasets. After internal validation with 500 bootstrap resamples, the optimism-corrected AUC was 0.807 and the bootstrap-derived calibration slope was 0.853. The final model is given as follows: logit[P(ROSC=1)] = −2.65−0.002 × age−0.790 × gender + 0.076 × white blood cell count + 0.001 × platelet count + 0.010 × PCO_2_−0.011 × admission glucose + 0.145 × fibrinogen−0.247 × serum potassium+0.633 × serum calcium.

**Conclusion:**

In patients who experienced OHCA, higher white blood cell count and lower serum potassium levels were associated with a higher incidence of sustained ROSC. Female patients were more likely to achieve sustained ROSC than male patients. Although the model showed certain predictive value for sustained ROSC, further multicenter, large-scale studies are needed in the future.

## 1 Introduction

Out-of-hospital cardiac arrest (OHCA) remains a major public health challenge globally, with substantial geographic variation in clinical outcomes. An aggregated analysis from the International Liaison Committee on Resuscitation (ILCOR) international registry reported that survival to hospital discharge or 30-day survival rates ranged from 3.1% to 20.4% across regions (1). Similarly, a prior systematic review (2) has demonstrated marked regional differences in OHCA incidence and survival rates, although the overall survival rate remains limited. These findings highlight the need for timely and reliable risk stratification during resuscitation, particularly using information that can be obtained early in the course of cardiopulmonary resuscitation (CPR). Global ischemia–reperfusion following cardiac arrest can rapidly precipitate acid–base derangements, electrolyte disturbances, coagulopathy, and metabolic abnormalities (3, 4). Blood samples obtained during resuscitation provide an objective assessment of these physiologic perturbations and may therefore support early return of spontaneous circulation (ROSC) risk stratification.

However, existing ROSC prediction models have limited applicability for real-time intra-arrest risk stratification because many of them rely on variables that are unavailable or can be obtained only later in the resuscitation process, and external validation remains limited (5, 6). Laboratory markers obtained during CPR may provide objective physiologic information at an early stage, but their predictive value for sustained ROSC has not been well established. The credibility of prediction models also depends on appropriate handling of missing data, control of overfitting, and transparent assessment of discrimination and calibration (7). In OHCA cohorts, missingness in laboratory variables is common, and multiple imputation by chained equations (MICE) can improve statistical efficiency under appropriate missing data assumptions (8).

Accordingly, in this study, we aimed to develop and internally validate a blood marker–based multivariable prediction model for sustained ROSC in a real-world cohort of patients who experienced OHCA and consequently were admitted to the emergency department using laboratory variables obtained during ongoing CPR together with prespecified demographic covariates.

## 2 Methods

### 2.1 Study design

This is a retrospective study conducted between January 2021 and December 2025 in a cohort of patients who experienced OHCA and presented to the emergency resuscitation room of a tertiary hospital in Zhejiang Province, China. Demographic and clinical data and CPR outcomes were retrospectively collected. The inclusion criteria were as follows: (1) age ≥18 years, (2) out-of-hospital cardiac arrest with continued cardiopulmonary resuscitation after emergency department admission, and (3) blood specimens collected during ongoing CPR in the emergency department for routine laboratory testing. No additional exclusion criteria were applied. This study has been approved by the hospital's ethics committee (approval no. (2024) Lun Shen Yan No. (1292)). No formal *a priori* sample size calculation was performed because of the retrospective design of this study. All consecutively eligible patients who experienced OHCA and met the predefined inclusion criteria during the study period were included.

### 2.2 Data collection

The predictor measurement time point was defined as the first blood sampling performed during CPR in the emergency department, which is done before determining the final resuscitation outcome. Laboratory variables included complete blood count indices, inflammatory markers, coagulation parameters, cardiac and metabolic markers, electrolytes, and blood gas variables. Blood gas variables included pH, PCO_2_, PO_2_, base excess, bicarbonate, and lactate. Because the blood gas sampling site was not systematically documented in the retrospective records, arterial, venous, and mixed samples could not be reliably distinguished; therefore, PCO_2_ is reported throughout the manuscript as blood PCO_2_ measured during CPR rather than as PaCO_2_. All laboratory measurements were based on the first available result obtained during the CPR. All samples were analyzed using standardized analytical instruments to ensure accuracy of the data. The primary outcome was sustained circulation for ≥20 min after cessation of chest compressions. This represented sustained ROSC in patients who received conventional CPR; patients who received extracorporeal CPR (ECPR) were included in the primary analysis, and a sensitivity analysis excluding all patients who received ECPR was performed to evaluate the potential influence of extracorporeal membrane oxygenation–supported circulation on the findings. Because of the retrospective design of this study, no formal blinding procedure was implemented. Laboratory predictors were objectively measured during the CPR before determination of the final resuscitation outcome, and the outcome was retrospectively identified from resuscitation records.

### 2.3 Statistical analyses

Continuous variables are summarized as mean ± SD for approximately normally distributed data and as median (25th, 75th percentiles) for non-normally distributed data; categorical variables are presented as counts (percentages). Missing data were handled using MICE, generating 20 imputed datasets (*m* = 20). Predictive mean matching (PMM) was used for continuous variables, logistic regression imputation was applied for binary variables, and polytomous (multinomial) logistic regression imputation was used for nominal variables with more than two categories. Sustained ROSC status was fully observed and was not imputed, but it was included as a predictor in the imputation models for variables with missing values. Therefore, the imputation procedure assumed that data were missing at random conditional on the observed variables included in the imputation model. Twenty iterations were performed for the imputation procedure, and a prespecified random seed was used to ensure reproducibility. Group comparisons stratified by sustained ROSC status (sustained ROSC vs. non-ROSC) were presented. For continuous variables, between-group comparisons were performed using the independent samples *t*-test for approximately normally distributed variables and the Mann–Whitney *U* test for non-normally distributed variables. For categorical variables, differences between groups were assessed using the chi-square test or Fisher's exact test, as appropriate. For continuous variables analyzed after multiple imputation, *P*-values were pooled across the imputed datasets using Rubin's rules (9). Variables with >50% missingness were described only descriptively and were not subjected to inferential testing. For subgroup analyses, non-imputed data were dichotomized according to trauma status, presumed etiology of cardiac arrest (classified using the 5H/5T framework), and each laboratory indicator; within each subgroup, differences between the sustained ROSC and non-ROSC groups were evaluated and visualized using heatmaps.

Candidate predictors for multivariable modeling were identified based on clinical relevance, previous literature, and their availability at the intended prediction time point. Age and gender were prespecified as clinically relevant demographic covariates and were retained irrespective of their univariable statistical significance. For laboratory variables, a univariable analysis was used as an additional screening step, with *P* < 0.05 considered together with clinical plausibility, prior evidence, redundancy among related biomarkers, and model parsimony. Variables representing treatment processes or information accumulated after the intended prediction time point, including total chest compression duration and cumulative epinephrine dose, were not considered eligible candidate predictors.

Multivariable analyses were performed using logistic regression after multiple imputation. Gender was coded as female = 0 and male = 1 in the multivariable model. Specifically, a binomial logistic regression model was fitted separately within each imputed dataset, and regression coefficients, standard errors, *P*-values, and 95% confidence intervals were combined using Rubin's rules (9); odds ratios (ORs) with corresponding 95% confidence intervals are reported.

Bootstrap internal validation was subsequently performed using the fixed final predictor set. Predictor selection was not repeated within each bootstrap resample. Thus, the bootstrap procedure quantified optimism conditional on the selected final model but did not incorporate additional optimism that may have arisen from the preceding predictor selection process. Within each imputed dataset, 500 bootstrap resamples were generated (10). Optimism was estimated as the mean difference between the apparent area under the curve (AUC) evaluated in each bootstrap sample and the corresponding test AUC evaluated in the original imputed dataset, and an optimism-corrected AUC was derived accordingly. Because predictor selection was not incorporated into the bootstrap resampling procedure, the corrected AUC may retain some residual optimism and should therefore be interpreted cautiously.

Model calibration was assessed using the calibration slope and calibration-in-the-large (CIL). A uniform shrinkage factor was derived from the mean bootstrap calibration slope; when indicated, regression coefficients were uniformly shrunk and the intercept was recalibrated to obtain the final shrunken model.

Model discrimination was additionally visualized using a receiver operating characteristic (ROC) curve, and calibration was graphically assessed by comparing predicted probabilities with the observed sustained ROSC proportions. The ROC and calibration plots of the final model are presented in Figures 2, 3, respectively.

Prespecified sensitivity analyses were performed to assess the influence of potentially heterogeneous clinical subgroups and blood gas sampling uncertainty. First, the final fixed model was refitted after excluding blood PCO_2_. Second, the model was refitted after exclusion of patients who received ECPR. Third, patients who experienced traumatic OHCA were excluded, and the model was refitted in patients who experienced non-traumatic OHCA. The same fixed predictor set, except for the intentionally excluded variable or subgroup, was used in each sensitivity analysis. Model coefficients and discrimination were compared with those of the primary analysis. All statistical tests were two-sided, with *P* < 0.05 considered statistically significant. All analyses were conducted in R (R Foundation for Statistical Computing).

## 3 Results

### 3.1 Comparison of general information and laboratory test indicators between the sustained ROSC and non-ROSC groups

During the study period, a total of 283 patients who experienced OHCA were included—comprising 94 patients in the sustained ROSC group and 189 in the non-ROSC group—yielding an overall sustained ROSC rate of 33.22% (Figure 1). Patterns of missingness and the multiple imputation strategy are presented in Table 1. Missing data were handled using MICE, generating 20 imputed datasets, and inference was pooled according to Rubin's rules. The sustained ROSC group exhibited higher PCO_2_, white blood cell count, platelet count, and absolute lymphocyte count; higher admission capillary blood glucose, fibrinogen, and serum calcium levels; shorter chest compression duration and prothrombin time; and lower cumulative epinephrine dose, serum potassium level, and international normalized ratio (INR; all *P* < 0.05) than the non-ROSC group. Differences were also observed in the distribution of past medical history and history of malignancy between the two groups (*P* < 0.05; Table 2). Subgroup analyses are presented in Supplementary Figure S1.

**Figure 1:**
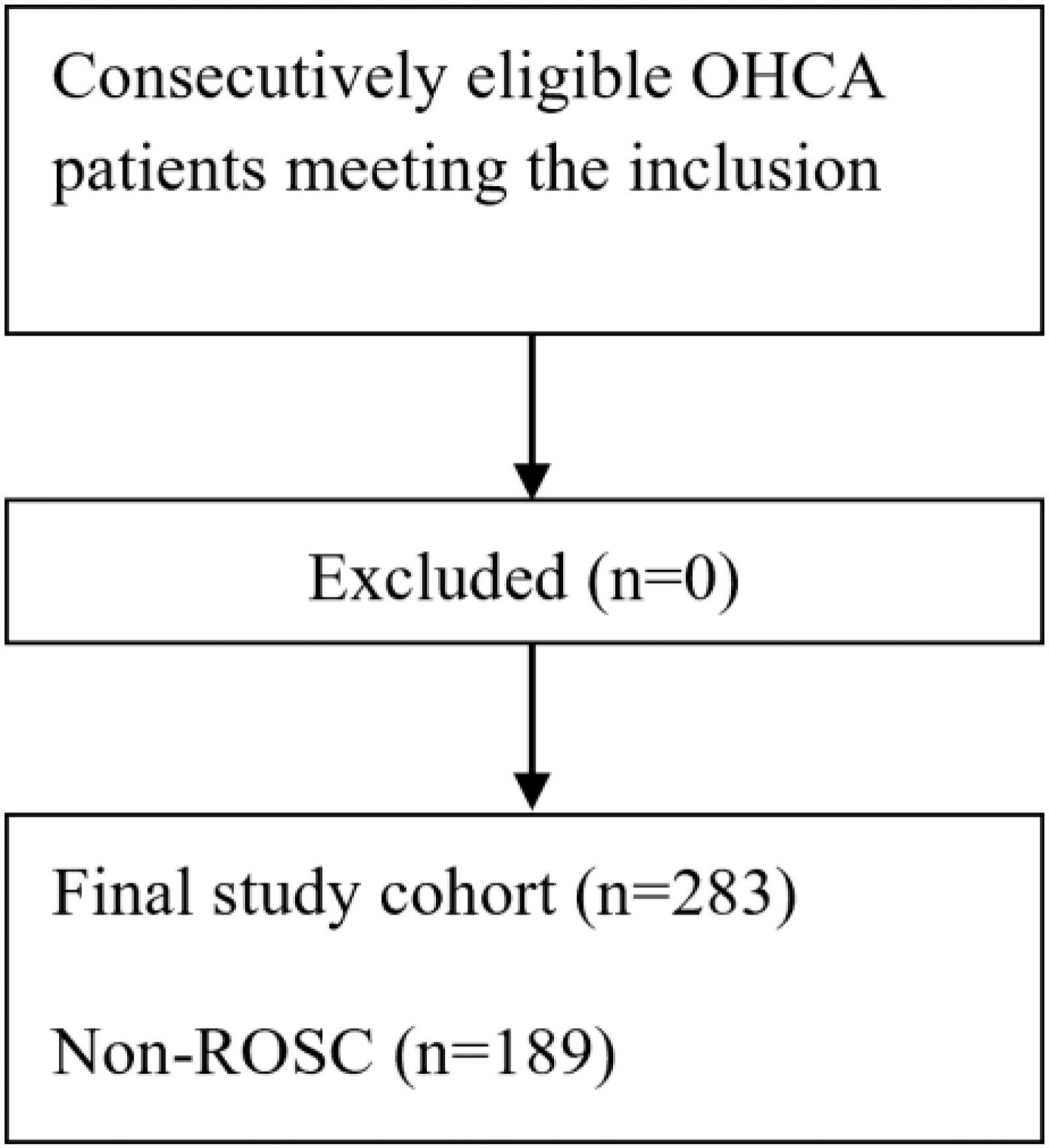
A patient flow diagram.

**Table 1:** Variables missingness and the multiple imputation strategy.

Variables	Missing (*n*)	Missing (%)	Method	Variables	Missing (*n*)	Missing (%)	Method
Age, years	0	0	PMM	Potassium, mmol/L	65	22.97	PMM
Capillary blood glucose on admission, mmol/L	9	3.18	PMM	Sodium, mmol/L	63	22.26	PMM
Chest compression duration, min	1	0.35	PMM	Chloride, mmol/L	195	68.9	PMM
Cumulative epinephrine dose, mL	2	0.71	PMM	Calcium, mmol/L	64	22.61	PMM
WBC, 10^9^/L	62	21.91	PMM	Blood glucose, mmol/L	62	21.91	PMM
RBC, 10^9^/L	62	21.91	PMM	Urea nitrogen, mmol/L	72	25.44	PMM
Hemoglobin, g/L	62	21.91	PMM	Creatinine, µmol/L	69	24.38	PMM
Hematocrit, L/L	60	21.2	PMM	Ph	106	37.46	PMM
Erythrocyte distribution width, %	63	22.26	PMM	PO_2_, mmHg	78	27.56	PMM
Platelet, 10^9^/L	63	22.26	PMM	PCO_2_, mmHg	80	28.27	PMM
platelet distribution width, %	196	69.26	PMM	base excess, mmol/L	111	39.22	PMM
Absolute counts of lymphocyte, 10^9^/L	64	22.61	PMM	Bicarbonate, mmol/L	112	39.58	PMM
Absolute counts of neutrophil, 10^9^/L	64	22.61	PMM	Lactate, mmol/L	88	31.1	PMM
CRP, mg/L	76	26.86	PMM	Gender	0	0	logreg
Interleukin, pg/mL	218	77.03	PMM	Trauma	0	0	logreg
Heparin-binding protein, ng/mL	265	93.64	PMM	Cause of cardiac arrest	0	0	polyreg
Procalcitonin, ng/mL	233	82.33	PMM	Past medical history	0	0	logreg
Prothrombin time, s	76	26.86	PMM	History of neurological diseases	0	0	logreg
INR	89	31.45	PMM	History of neurological diseases	0	0	logreg
APTT, s	77	27.21	PMM	History of circulatory system diseases	0	0	logreg
Thrombin time, s	82	28.98	PMM	History of respiratory diseases	0	0	logreg
Fibrinogen, g/L	78	27.56	PMM	History of digestive diseases	0	0	logreg
D-dimer, µg/L	78	27.56	PMM	History of urinary diseases	0	0	logreg
BNP, pg/mL	203	71.73	PMM	History of immune system diseases	0	0	logreg
Total bilirubin, µmol/L	79	27.92	PMM	History of metabolic disease	0	0	logreg
Albumin, g/L	82	28.98	PMM	History of motor system diseases	0	0	logreg
Glutamic-pyruvic transaminase, U/L	85	30.04	PMM	Tumor	0	0	logreg
Creatine kinase, U/L	79	27.92	PMM	Others	0	0	logreg
Glutamic oxaloacetic transaminase, U/L	74	26.15	PMM	History of circulatory system diseases	0	0	logreg
cn-T, ng/mL	79	27.92	PMM	History of respiratory diseases	0	0	logreg

ROSC, return of spontaneous circulation; RBC, red blood cell; WBC, white blood cell; CRP, C-reaction protein; APTT, activated partial thromboplastin time; INR, international normalized ratio; BNP, B-type natriuretic peptide; PMM, predictive mean matching; logreg, logistic regression; polyreg, polytomous logistic regression.

**Table 2:** Comparison of clinical characteristics and intra-arrest blood markers between the ROSC and non-ROSC groups (*n* = 283).

Variables	Non-ROSC (*n* = 189)	ROSC (*n* = 94)	*t*/*Z*	*p*
Age, years	69.00 (53.00, 80.00)	67.50 (56.00, 84.00)	0.729	0.466
Capillary blood glucose on admission, mmol/L	8.30 (5.80, 13.80)	12.65 (8.80, 17.40)	2.900	**0**.**004**
Chest compression duration, min	46.00 (34.00, 63.00)	13.00 (8.00, 30.00)	−10.670	**0**.**000**
Cumulative epinephrine dose, mL	16.00 (12.00, 21.00)	5.00 (3.00, 10.00)	−9.412	**0**.**000**
WBC, 10^9^/L	8.10 (5.70, 11.80)	11.15 (8.20, 15.00)	4.695	**0**.**000**
RBC, 10^9^/L	3.62 (2.72, 4.43)	3.83 (3.01, 4.61)	1.572	0.118
Hemoglobin, g/L	112.00 (84.00, 136.00)	114.50 (91.00, 136.00)	1.260	0.209
Hematocrit, L/L	0.35 (0.27, 0.43)	0.35 (0.28, 0.44)	1.539	0.126
Erythrocyte distribution width, %	13.50 (12.80, 14.40)	13.80 (12.90, 15.40)	1.869	0.064
Platelet, 10^9^/L	104.00 (74.00, 164.00)	147.00 (103.00, 203.00)	3.575	**0**.**000**
Platelet distribution width, %	0.14 (0.09, 0.21)	0.18 (0.12, 0.22)	–	–
Absolute counts of lymphocyte, 10^9^/L	3.01 (1.84, 4.62)	4.54 (2.72, 6.08)	2.353	**0**.**020**
Absolute counts of neutrophil, 10^9^/L	4.07 (2.55, 6.59)	5.50 (3.60, 9.30)	1.464	0.147
CRP, mg/L	5.00 (1.10, 38.60)	15.45 (1.40, 88.40)	1.696	0.092
Interleukin, pg/mL	2,332.00 (116.00, 5,000.00)	1,885.00 (230.00, 5,000.00)	–	–
Heparin-binding protein, ng/mL	65.41 (23.45, 196.02)	81.21 (66.76, 108.06)	–	–
Procalcitonin, ng/mL	0.08 (0.04, 0.19)	0.30 (0.08, 0.55)	–	–
Prothrombin time, s	18.70 (15.10, 27.50)	16.65 (14.90, 20.90)	−2.111	**0**.**036**
INR	1.61 (1.18, 2.65)	1.38 (1.18, 1.80)	−2.268	**0**.**025**
APTT, s	54.30 (39.00, 97.60)	52.20 (40.90, 75.20)	−1.558	0.121
Thrombin time, s	19.20 (16.90, 27.20)	18.25 (16.40, 22.20)	−1.188	0.237
Fibrinogen, g/L	2.52 (1.14, 3.49)	3.11 (2.07, 4.50)	2.835	**0**.**005**
D-dimer, µg/L	20,000.00 (6,200.00, 20,000.00)	14,420.00 (4,690.00, 20,000.00)	−1.696	0.092
BNP, pg/mL	1,295.00 (92.00, 7,015.00)	2,460.00 (129.00, 7,261.00)	–	–
Total bilirubin, µmol/L	14.40 (10.00, 22.50)	14.60 (9.30, 21.70)	0.600	0.549
Albumin, g/L	32.60 (27.40, 39.50)	34.00 (28.70, 37.70)	0.750	0.454
Glutamic–pyruvic transaminase, U/L	50.00 (29.00, 126.00)	38.50 (22.00, 118.00)	1.235	0.218
Creatine kinase, U/L	208.00 (116.00, 518.00)	185.00 (90.00, 394.00)	0.451	0.652
Glutamic oxaloacetic transaminase, U/L	85.00 (47.00, 247.00)	83.00 (48.00, 265.00)	1.764	0.079
cn-T, ng/mL	0.06 (0.02, 0.15)	0.06 (0.03, 0.13)	0.336	0.737
Potassium, mmol/L	5.54 (4.21, 7.29)	5.00 (4.18, 6.60)	−2.628	**0**.**009**
Sodium, mmol/L	143.70 (140.10, 147.80)	143.05 (138.90, 146.70)	−1.957	0.052
Chloride, mmol/L	107.60 (102.20, 123.80)	103.95 (97.20, 111.40)	–	–
Calcium, mmol/L	2.10 (1.67, 2.27)	2.10 (1.93, 2.22)	2.038	**0**.**043**
Laboratory blood glucose, mmol/L	8.60 (5.16, 13.94)	12.87 (9.17, 17.65)	2.563	**0**.**011**
Urea nitrogen, mmol/L	6.61 (4.94, 9.26)	6.87 (4.79, 11.54)	0.369	0.712
Creatinine, µmol/L	102.00 (76.00, 138.70)	113.00 (82.00, 158.00)	1.276	0.203
pH	7.02 (6.88, 7.10)	6.97 (6.90, 7.09)	−0.749	0.454
PO_2_, mmHg	72.40 (51.70, 94.20)	70.95 (47.60, 97.10)	−0.898	0.371
PCO_2_, mmHg	28.30 (16.20, 58.40)	112.00 (51.10, 218.00)	5.709	**0**.**000**
Base excess, mmol/L	−16.20(−22.00, −11.00)	−16.35(−21.00, −11.70)	−0.207	0.836
Bicarbonate, mmol/L	16.70 (11.40, 20.30)	15.85 (10.40, 20.30)	0.084	0.933
Lactate, mmol/L	13.50 (8.90, 19.00)	13.70 (10.60, 18.00)	0.033	0.974
Female, *n* (%)	48 (25.40)	33 (35.11)	2.897	0.089
Trauma, *n* (%)	142 (75.13)	83 (88.30)	6.678	**0**.**001**
Cause of cardiac arrest			–	0.156^a^
Unknown, *n* (%)	11 (5.82)	2 (2.13)		
Hypoxia, *n* (%)	60 (31.75)	43 (45.74)		
Hypovolemia, *n* (%)	44 (23.28)	15 (15.96)		
Acidosis, *n* (%)	11 (5.82)	4 (4.26)		
Hypoglycemia, *n* (%)	1 (0.53)	1 (1.06)		
Hyperkalemia/Hypokalemia, *n* (%)	16 (8.47)	10 (10.64)		
Cardiac tamponade, *n* (%)	5 (2.65)	0 (0.00)		
Pulmonary embolism, *n* (%)	2 (1.06)	2 (2.13)		
Tension pneumothorax, *n* (%)	3 (1.59)	0 (0.00)		
Acute myocardial infarction, *n* (%)	36 (19.05)	16 (17.02)		
Intracranial cause, *n* (%)	0 (0.00)	1 (1.06)		
Past medical history, *n* (%)	103 (54.50)	69 (73.40)	9.414	**0**.**002**
History of neurological diseases, *n* (%)	16 (8.47)	15 (15.96)	3.612	0.057
History of circulatory system diseases, *n* (%)	64 (33.86)	36 (38.30)	0.541	0.462
History of respiratory diseases, *n* (%)	13 (6.88)	9 (9.57)	0.637	0.425
History of digestive diseases, *n* (%)	9 (4.76)	3 (3.19)	0.093	0.761
History of Urinary diseases, *n* (%)	4 (2.12)	3 (3.19)	0.020	0.887
History of immune system diseases, *n* (%)	0 (0.00)	1 (1.06)	–	0.332^a^
History of metabolic disease, *n* (%)	23 (12.17)	19 (20.21)	3.214	0.073
History of motor system diseases, *n* (%)	3 (1.59)	6 (6.38)	3.261	0.071
Tumor, *n* (%)	20 (10.58)	20 (21.28)	5.916	**0**.**015**
History of other diseases, *n* (%)	4 (2.12)	0 (0.00)	0.785	0.376

ROSC, return of spontaneous circulation; RBC, red blood cell; WBC, white blood cell; CRP, C-reactive protein; APTT, activated partial thromboplastin time; INR, international normalized ratio; BNP, B-type natriuretic peptide.

Continuous data are expressed as median (25th–75th percentiles) for non-normally distributed data. Categorical variables are expressed as counts (percentage). The values in bold indicate statistical significance.

a Fisher's exact test.

### 3.2 Multivariable logistic regression analysis after multiple imputation

Multivariable logistic regression models were fitted separately within each of the 20 imputed datasets, and regression coefficients and their standard errors were pooled using Rubin's rules. After inclusion of age, gender, white blood cell count, platelet count, and PCO_2_ and admission capillary blood glucose, fibrinogen, serum potassium, and serum calcium levels, the pooled results indicated that higher white blood cell count and higher PCO_2_ were associated with an increased probability of sustained ROSC, whereas higher serum potassium levels and male gender were associated with a decreased probability of sustained ROSC (Table 3).

**Table 3:** A pooled multivariable logistic regression model for ROSC after multiple imputation.

Variables	Estimate	OR	95% CI	*p*
(Intercept)	−2.116	0.121	(0.006–2.479)	0.172
Age, years	−0.003	0.997	(0.972–1.023)	0.836
WBC, ×10⁹/L	0.089	1.093	(1.020–1.171)	**0** **.** **013**
Platelet, ×10⁹/L	0.002	1.002	(0.997–1.007)	0.515
PCO_2_, mmHg	0.012	1.012	(1.007–1.017)	**0**.**000**
Capillary blood glucose on admission, mmol/L	−0.013	0.987	(0.942–1.033)	0.566
Fibrinogen, g/L	0.170	1.186	(0.959–1.467)	0.118
Potassium, mmol/L	−0.289	0.749	(0.600–0.934)	**0**.**011**
Calcium, mmol/L	0.741	2.099	(0.674–6.538)	0.203
Gender (male vs. female)	−0.925	0.396	(0.165–0.950)	**0**.**040**

Regression estimates were pooled across 20 multiply imputed datasets using Rubin's rules. Sex was coded as female = 0 and male = 1.

The values in bold indicate statistical significance (*P* < 0.05).

### 3.3 Internal validation and model performance

The fixed final model yielded a mean apparent AUC of 0.832 across the 20 imputed datasets. After bootstrap correction, the mean optimism-corrected AUC was 0.807. The bootstrap-derived mean calibration slope was 0.853, indicating some degree of overfitting, and the mean bootstrap calibration-in-the-large was −0.093, indicating slight overall overestimation of predicted probabilities (Table 4). Regression coefficients were subsequently uniformly shrunk, and the intercept was recalibrated to obtain the final model.

**Table 4:** Bootstrap internal validation of the fixed final model across 20 imputed datasets (500 resamples per dataset).

Metric	Mean	SD
Apparent AUC	0.832	0.018
Estimated optimism in AUC	0.025	0.002
Optimism-corrected AUC	0.807	0.020
Bootstrap-derived calibration slope/uniform shrinkage factor	0.853	0.011
Mean bootstrap calibration-in-the-large	−0.093	0.009
Recalibrated intercept after shrinkage	−2.653	0.372

Predictor selection was performed before bootstrap validation and was not repeated within each bootstrap resample. Therefore, the optimism-corrected AUC reflects internal validation conditional on the fixed final predictor set and may retain residual optimism related to predictor selection.

Model discrimination and calibration plots are shown in Figures 2, 3, respectively. Because predictor selection was performed before bootstrap validation and was not repeated within each bootstrap resample, the optimism-corrected performance estimates should be interpreted as conditional on the fixed final predictor set.

**Figure 2:**
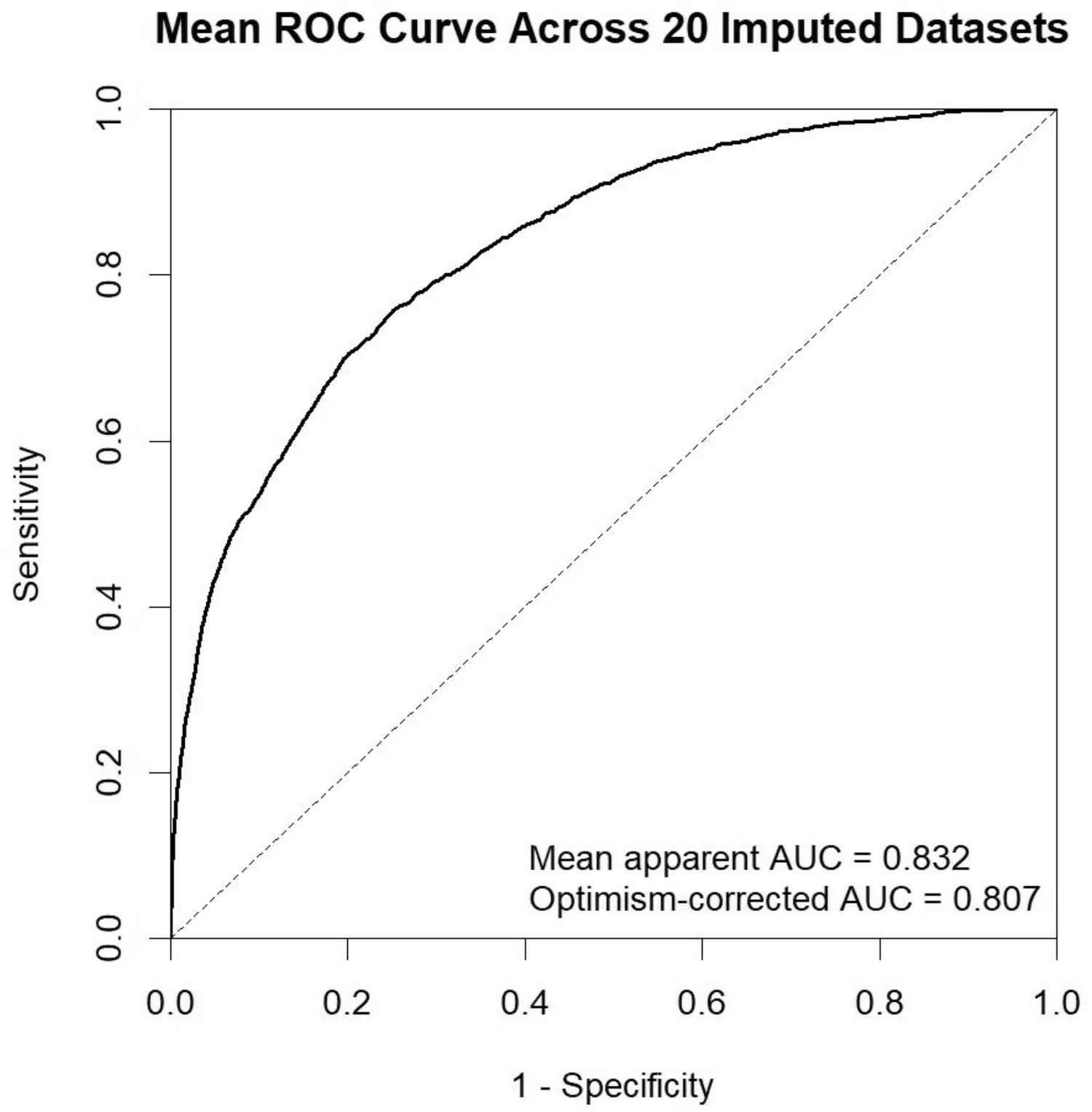
The mean receiver operating characteristic curve across the 20 multiply imputed datasets.

**Figure 3:**
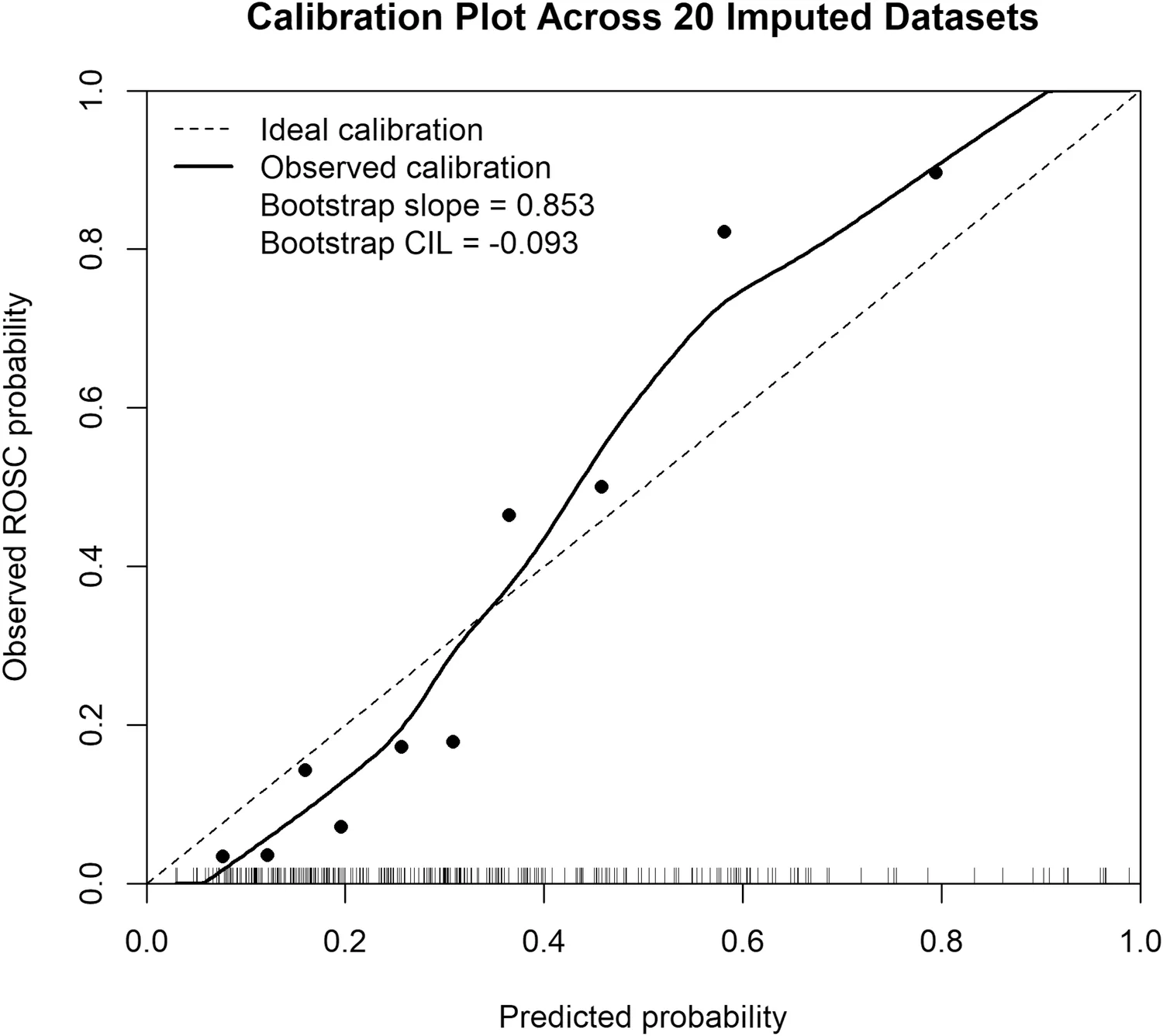
A calibration plot of the final ROSC prediction model across the 20 multiply imputed datasets.

### 3.4 Sensitivity analyses

After exclusion of blood PCO_2_, the optimism-corrected AUC decreased from 0.807 to 0.728. After exclusion of 16 patients who received ECPR, the optimism-corrected AUC was 0.841. After exclusion of 58 patients who experienced traumatic OHCA, the optimism-corrected AUC was 0.757. The direction of most predictor associations was preserved, although some coefficient estimates and statistical significance changed across sensitivity analyses. Detailed results are provided in Supplementary Tables S3–S8.

## 4 Discussion

We retrospectively analyzed physiologic laboratory markers obtained during emergency department CPR in 283 patients who experienced OHCA. Missing data were handled using multiple imputation (*m* = 20). A multivariable prediction model for sustained ROSC was developed and internally validated using bootstrap resampling.

Missingness varied across laboratory variables in this retrospective cohort. Variables with >50% missingness were retained for descriptive purposes only and were not included in inferential analyses or model development. For variables included in the analyses, missing data were handled using MICE with 20 imputed datasets. Sustained ROSC status was completely observed and was not imputed, but it was included as a predictor in the imputation models for variables with missing values. Missingness was additionally reported separately for the sustained ROSC and non-ROSC groups to allow assessment of outcome-related differences in data availability. Although inclusion of sustained ROSC and other observed variables in the imputation models allowed outcome-related missingness to be accommodated under the missing-at-random assumption, this assumption cannot be verified from the observed data alone. A residual missing-not-at-random mechanism related to unobserved laboratory values or unmeasured clinical factors therefore cannot be excluded.

The laboratory differences observed between patients with and without sustained ROSC should be interpreted collectively as markers of the systemic physiologic disturbances occurring during cardiac arrest and CPR rather than as isolated causal factors. Elevated leukocyte counts during resuscitation may partly reflect catecholamine-induced leukocyte demargination rather than infection alone (11, 12). Alterations in platelet count, prothrombin time, INR, and fibrinogen levels may reflect activation and consumption of the coagulation system during cardiac arrest and resuscitation (13–15). Similarly, elevated serum potassium levels may indicate more severe ischemia, acidosis-related transcellular shifts, and impaired myocardial excitability (16, 17). Hemodynamic responsiveness to epinephrine during CPR has also been associated with ROSC (18), further illustrating the dynamic physiologic changes occurring during resuscitation. Taken together, these findings suggest that the predictive information may arise from the combined physiologic profile during CPR rather than from any single biomarker.

Blood PCO_2_ requires particular caution. During CPR, its measured concentration may reflect a complex interaction among ventilation, systemic blood flow, tissue CO_2_ production and transport, and sampling source. In our retrospective dataset, neither the blood gas sampling site nor the timing of sampling relative to advanced airway placement and initiation of positive pressure ventilation could be reliably reconstructed. Accordingly, the association between blood PCO_2_ and sustained ROSC should not be interpreted as evidence of a specific arterial or ventilation-related mechanism. In the sensitivity analysis, exclusion of blood PCO_2_ reduced the optimism-corrected AUC from 0.807 to 0.728, indicating that model discrimination was partly dependent on this variable. With regard to gender, previous studies have reported inconsistent sex-related differences in OHCA outcomes, with some suggesting higher odds of ROSC among women and others showing attenuation of the association after adjustment for resuscitation-related factors (19, 20). Therefore, both blood PCO_2_ and gender should be interpreted as predictive covariates rather than as independent causal determinants of sustained ROSC.

Compared with previously reported ROSC prediction models, the present model differs mainly in its emphasis on laboratory information obtained during ongoing CPR. Existing models have commonly incorporated clinical and resuscitation variables such as initial rhythm, etiology, witnessed status, bystander CPR, resuscitation duration, age, and sex, while methodological limitations related to predictor selection, missing data handling, overfitting, and external validation have also been reported (5). In contrast, our model focused on intra-arrest laboratory markers, used multiple imputation for missing data, and assessed both discrimination and calibration employing bootstrap internal validation. This approach may provide complementary physiologic information during CPR. However, because core Utstein variables were not systematically available, the present study cannot establish whether these laboratory markers provide incremental predictive value beyond established clinical predictors. External validation incorporating both standardized Utstein variables and intra-arrest laboratory measurements is therefore required before clinical application.

The initial regression model yielded an apparent AUC of 0.832. After bootstrap correction, the optimism-corrected AUC was 0.807. The bootstrap-derived mean calibration slope was approximately 0.853, suggesting some degree of overfitting with overly extreme predicted probabilities. In addition, the bootstrap-derived CIL was −0.093, indicating slight overall overestimation of the predicted risk. Based on these findings, we applied uniform shrinkage and intercept recalibration to improve the reliability of predicted probabilities. The final model is given as follows: logit[P(ROSC=1)] = −2.65−0.002 × age−0.790 × gender + 0.076 × white blood cell count + 0.001 × platelet count + 0.010 × PCO_2_−0.011 × admission glucose + 0.145 × fibrinogen−0.247 × serum potassium + 0.633 × serum calcium. Performance estimates were relatively consistent across the 20 imputed datasets; however, this consistency does not exclude bias arising from violations of the missing-at-random assumption. Nevertheless, because these results are based on internal validation, external validation in independent cohorts is required to confirm transportability and calibration stability.

Nevertheless, several limitations of this study should be acknowledged. First, this was a single-center retrospective study, which may limit its representativeness and generalizability. Although all consecutively eligible patients were included and no patients were excluded because of missing study variables, selection bias related to the predefined eligibility criteria and availability of blood sampling during ongoing CPR cannot be completely excluded. Second, varying degrees of missingness were present across laboratory variables. Although MICE incorporated sustained ROSC status and other observed variables, the missing-at-random assumption cannot be verified, and a residual missing-not-at-random mechanism cannot be excluded. Third, the blood gas sampling site could not be retrospectively verified for all patients, and therefore, some PCO_2_ measurements may have originated from venous or mixed samples. In the sensitivity analysis excluding PCO_2_, the optimism-corrected AUC decreased from 0.807 to 0.728, indicating that model discrimination was partly dependent on this variable. Accordingly, the predictive contribution of blood PCO_2_ should be interpreted cautiously. Fourth, this study was specifically designed to evaluate the predictive information obtained from blood markers during CPR, and several core Utstein variables, including initial rhythm, witnessed status, bystander CPR, and no-flow/low-flow times, were not systematically collected. Therefore, the present study cannot determine whether the identified blood markers provide incremental predictive value beyond established Utstein predictors. Fifth, sensitivity analyses showed that exclusion of patients who received ECPR did not materially attenuate model performance, whereas exclusion of patients who experienced traumatic OHCA reduced the optimism-corrected AUC from 0.807 to 0.757, suggesting some heterogeneity between traumatic and non-traumatic OHCA. Finally, predictor selection was performed before bootstrap internal validation and was not repeated within each bootstrap resample. Consequently, although bootstrap correction was applied to the fixed final model, optimism introduced during the preceding predictor selection process may not have been fully captured. The reported optimism-corrected AUC may therefore remain somewhat optimistic. External validation in independent cohorts incorporating standardized Utstein variables and blood sampling procedures is warranted.

## 5 Conclusion

In this retrospective OHCA cohort, a blood marker–based model using laboratory variables obtained during CPR demonstrated potential in discriminating patients who achieved sustained ROSC from those who did not. White blood cell count, serum potassium level, gender, and blood PCO_2_ were associated with sustained ROSC in the multivariable model. However, model performance was partly dependent on blood PCO_2_, and uncertainty regarding the blood gas sampling source, the absence of core Utstein variables, heterogeneity between traumatic and non-traumatic OHCA, and residual optimism related to predictor selection limit immediate clinical application. Prospective external validation incorporating standardized Utstein variables and standardized blood gas sampling is required.

## Data Availability

The original contributions presented in the study are included in the article/Supplementary Material, further inquiries can be directed to the corresponding author.
