# Supplementary material for: The predictive model of intra-arrest blood markers for the sustained return of spontaneous circulation in patients with out-of-hospital cardiac arrest
Source: Front Med (Lausanne). 2026 Oct 13;13:1904268. doi: 10.3389/fmed.2026.1904268 (PMC13710292; doi:10.3389/fmed.2026.1904268)
Supplement: Supplementary file 1 [file Table1.docx]

| **Section/Topic** | **Item** | **Checklist Item** | **Page/Section where reported** |
| --- | --- | --- | --- |
| **Title and abstract** |  |  |  |
| Title | 1 | Identify the study as developing and/or validating a multivariable prediction model, the target population, and the outcome to be predicted. | **Page 1, Title** |
| Abstract | 2 | Provide a summary of objectives, study design, setting, participants, sample size, predictors, outcome, statistical analysis, results, and conclusions. | **Pages 1–2, Abstract** |
| **Introduction** |  |  |  |
| Background and objectives | 3a | Explain the medical context and rationale for developing the multivariable prediction model, including references to existing models. | **Page 2, Introduction** |
| Background and objectives | 3b | Specify the objectives, including whether the study describes the development or validation of the model or both. | **Page 2, Introduction, final paragraph** |
| **Methods** |  |  |  |
| Source of data | 4a | Describe the study design or source of data. | **Page 2–3, Methods 1.1 Study design** |
| Source of data | 4b | Specify key study dates, including start and end of accrual and, if applicable, end of follow-up. | **Page 2–3, Methods 1.1 Study design** |
| Participants | 5a | Specify key elements of the study setting, including number and location of centres. | **Page 3, Methods 1.1 Study design** |
| Participants | 5b | Describe eligibility criteria for participants. | **Page 3, Methods 1.1 Study design** |
| Participants | 5c | Give details of treatments received, if relevant. | **Pages 3–5, Methods 1.1–1.3; CPR/ECPR described where relevant** |
| Outcome | 6a | Clearly define the outcome predicted by the model, including how and when it was assessed. | **Page 3, Methods 1.2 Data collection** |
| Outcome | 6b | Report any actions to blind assessment of the outcome to be predicted. | **Page 3, Methods 1.2 Data collection** |
| Predictors | 7a | Clearly define all predictors used in model development, including how and when they were measured. | **Pages 3–5, Methods 1.2–1.3; Table 3** |
| Predictors | 7b | Report any actions to blind assessment of predictors for the outcome and other predictors. | **Page 3, Methods 1.2 Data collection** |
| Sample size | 8 | Explain how the study size was arrived at. | **Page 3, Methods 1.1 Study design** |
| Missing data | 9 | Describe how missing data were handled, with details of any imputation method. | **Pages 3–4, Methods 1.3 Statistical analyses; Table 1** |
| Statistical analysis methods | 10a | Describe how predictors were handled in the analyses. | **Page 4, Methods 1.3 Statistical analyses** |
| Statistical analysis methods | 10b | Specify the type of model, all model-building procedures, including predictor selection, and method for internal validation. | **Pages 4–5, Methods 1.3 Statistical analyses** |
| Statistical analysis methods | 10d | Specify all measures used to assess model performance and, if relevant, compare multiple models. | **Pages 4–5, Methods 1.3 Statistical analyses** |
| Risk groups | 11 | Provide details on how risk groups were created, if done. | **N/A – no risk groups were created** |
| **Results** |  |  |  |
| Participants | 13a | Describe the flow of participants through the study, including the number with and without the outcome; a diagram may be helpful. | **Page 5, Results 2.1; Figure 1** |
| Participants | 13b | Describe participant characteristics, including available predictors and the number of participants with missing predictor/outcome data. | **Page 5, Results 2.1; Tables 1–2** |
| Model development | 14a | Specify the number of participants and outcome events in each analysis. | **Pages 5–6, Results 2.1–2.4; Supplementary Tables S3–S8** |
| Model development | 14b | If done, report the unadjusted association between each candidate predictor and outcome. | **Page 5, Results 2.1; Table 2** |
| Model specification | 15a | Present the full prediction model to allow predictions for individuals, including all regression coefficients and intercept. | **Page 1, Abstract; Page 8–9, Discussion; Table 3** |
| Model specification | 15b | Explain how to use the prediction model. | **Model equation provided in Abstract and Discussion; individual predicted probability can be derived from the reported logistic equation** |
| Model performance | 16 | Report performance measures for the prediction model. | **Page 6, Results 2.3; Table 4; Figures 2–3** |
| **Discussion** |  |  |  |
| Limitations | 18 | Discuss any limitations of the study, such as nonrepresentative sample, few events per predictor, and missing data. | **Pages 9–10, Discussion, Limitations** |
| Interpretation | 19b | Give an overall interpretation of the results, considering objectives, limitations, results from similar studies, and other relevant evidence. | **Pages 6–10, Discussion** |
| Implications | 20 | Discuss the potential clinical use of the model and implications for future research. | **Pages 9–10, Discussion and Conclusion** |
| **Other information** |  |  |  |
| Supplementary information | 21 | Provide information about the availability of supplementary resources, such as study protocol, Web calculator, and datasets. | **Supplementary Figure S1 and Supplementary Tables S3–S8 are provided with the manuscript** |
| Funding | 22 | Give the source of funding and the role of the funders for the present study. | **No external funding was received for this study.** |
